# Managing global risks of invasive aquatic plants under climate change: ensemble species distribution modeling and economic cost projections

**DOI:** 10.3389/fpls.2026.1895896

**Published:** 2026-09-07

**Authors:** Wei Xing, Zixuan Feng, Wenmin Huang, Xiaowei Li

**Affiliations:** 1 Key Laboratory of Intelligent Health Perception and Ecological Restoration of Rivers and Lakes, Ministry of Education, Hubei University of Technology, Wuhan, China; 2 Innovation Demonstration Base of Ecological Environment Geotechnical and Ecological Restoration of Rivers and Lakes, Hubei University of Technology, Wuhan, China; 3 State Key Laboratory of Lake and Watershed Science for Water Security, Wuhan Botanical Garden, Chinese Academy of Sciences, Wuhan, China; 4 Hubei Key Laboratory of Environmental Geotechnology and Ecological Remediation for Lake and River, Hubei University of Technology, Wuhan, China; 5 School of Civil Engineering, Architecture and Environment, Hubei University of Technology, Wuhan, China; 6 Central - Southern Safety and Environment Technology Institute Co. Ltd., Wuhan, China

**Keywords:** climate change, economic losses, invasive aquatic plants, species distribution models, suitable areas

## Abstract

**Introduction:**

Invasive aquatic plants obstruct waterways, disrupt fisheries, and cause significant economic, social, and ecological impacts. Predicting their potential distribution and associated economic losses under climate change is critical for global risk management.

**Methods:**

This study aimed to predict the global potential distribution of four major invasive aquatic plants (*Pontederia crassipes, Pistia stratiotes, Cabomba caroliniana, Alternanthera philoxeroides*) and assess associated economic losses under current and future climates using species distribution modeling (Biomod2) and the Invacost database.

**Results:**

Current high‑suitability habitats for *P. crassipes* and *P. stratiotes* span South Asia, Africa, Europe, Oceania, and the Americas; *A. philoxeroides* concentrates in South Asia, the southeastern US, South America, and parts of Europe/Africa; C. caroliniana suitability is more limited to South/Southeast Asia and southeastern Americas. Future projections indicate significant northward expansion for all species, with *P. crassipes* and *P. stratiotes* experiencing the largest habitat increases (9.99% and 10.21%, respectively). Key environmental drivers include mean annual temperature, population density, and coldest season temperature. Global economic losses from 1990–2025 are estimated at $7,380.30 million (average $205.01 million/year), showing a rising trend.

**Discussion:**

This work provides critical insights for global monitoring, early detection, rapid response, and strategic management of invasive aquatic plants, offering theoretical support for invasion biology under climate change and informing policy decisions and public awareness efforts.

## 1 Introduction

Biological invasions, as one of the core threats of global change, have resulted in sharp declines in biodiversity, degradation of ecosystem services, and increased risks to human health (Mack et al., 2000; Oficialdegui et al., 2023). Invasive species contribute to the loss of genetic diversity and the decline in the value of ecosystem services by outcompeting native species, disrupting ecological functions, and causing widespread ecological damage (Carneiro et al., 2024). The direct economic impacts of biological invasions span across multiple sectors, including agriculture, infrastructure, and public health (Vantarová et al., 2023). Despite the annual global economic losses caused by biological invasions being estimated in the trillions of dollars (Diagne et al., 2021a), current management strategies remain heavily reliant on inefficient post-invasion control measures rather than proactive prevention (Beaury et al., 2020; Pyšek et al., 2020). Climate change and globalization have further exacerbated this crisis. Rising global temperatures are driving latitudinal shifts in species distributions and altering phenological patterns (Scheffers et al., 2016), while trade expansion and human mobility are providing new pathways for the spread of invasive species (Hulme, 2017). Notably, the destructive impacts of invasive aquatic plants on freshwater ecosystems have emerged as a global challenge. These species alter the physicochemical properties of water bodies and reduce community productivity, resulting in biodiversity loss and genetic homogenization (Cuthbert et al., 2021; Macêdo et al., 2024). Representative invaders including *Pontederia crassipes* and *Pistia stratiotes* obstruct waterways and water supply facilities and degrade fishery ecosystems. *P. crassipes* roots harbor pathogens, whereas *Alternanthera philoxeroides* canopies offer ideal breeding habitats for mosquitoes. These species induce extensive global economic losses across agriculture, fisheries, water transport, hydropower, and irrigation sectors. Given these escalating threats, there is an urgent need to predict where invasive species are most likely to establish and spread.

In this context, Species Distribution Models (SDMs) have become essential tools for predicting invasion hotspots and assessing risk levels (Hui, 2023; Li et al., 2023). Species Distribution Models, as core tools in ecology and biogeography, have been widely applied to predict the potential suitable habitats of invasive species, assess invasion risks, and analyze dispersal trends (Guisan et al., 2014). Among these, the Maximum Entropy Model (MaxEnt) has emerged as one of the most popular tools due to its efficient algorithm based on the principle of maximum entropy and its user-friendly interface (Elith et al., 2011; Merow et al., 2013). To further enhance prediction accuracy and stability, the ensemble modeling framework Biomod2 incorporates statistical methods and machine learning algorithms, significantly improving the reliability and robustness of predictions through model integration (Araújo and New, 2007). Studies have demonstrated that most invasive species exhibit climatic niche conservatism between their native and invaded ranges (Guareschi et al., 2024; Liu et al., 2020).

Notably, the application of SDMs in studies of invasive aquatic plants can reveal distribution dynamics and driving mechanisms (Vythalingam et al., 2022). Global analyses indicate that the suitable habitat for *Iris pseudacorus* in North America and East Asia is expected to expand northward, while suitable areas in the Southern Hemisphere are likely to contract (Minuti et al., 2023). The South-to-North Water Diversion Project may further accelerate their northward spread (Liu et al., 2017; Zhu et al., 2023). The integration of ensemble modeling frameworks, such as Biomod2, has significantly improved prediction accuracy. For example, the invasion risk of *P. crassipes* in Portugal’s Cavado River has been shown to be strongly correlated with the intensity of human activities (Mouta et al., 2023). However, these studies have focused primarily on individual species or regions, and few have integrated habitat suitability predictions with economic impact assessments. Meanwhile, the economic burden of invasive aquatic plants remains insufficiently quantified. As research in this field has advanced, the Invacost database has become a central tool for assessing the economic impacts of invasions, revealing that global costs reached USD 1.288 trillion between 1970 and 2017 (Diagne et al., 2021b; Fantle-Lepczyk et al., 2022), though these figures are likely underestimated due to geographic and taxonomic data gaps. However, current assessments predominantly focus on terrestrial species, whereas aquatic plants and other underrepresented taxonomic groups still require systematic study (Cuthbert et al., 2021; Macêdo et al., 2024).

To fill this gap, this study integrates ensemble SDMs (Biomod2) with the InvaCost database to jointly examine the habitat suitability and economic impacts of four globally widespread invasive aquatic plants: *Pontederia crassipes*, *Pistia stratiotes*, *Alternanthera philoxeroides*, and *Cabomba caroliniana*. We employed an integrated dataset to predict current and future suitable habitats of these species, enabling risk assessment and clarifying the influence of climate change and human activities on distributional shifts. By leveraging the Invacost database, this study also quantifies the global economic impacts of invasive aquatic plants through systematic analysis of temporal trends and spatial patterns.

Specifically, our objectives are to: (1) predict current global habitat suitability and identify the key environmental drivers for each of the four species; (2) project future distributional shifts under three Shared Socioeconomic Pathways (SSP126, SSP245, SSP585) across multiple time horizons; and (3) quantify historical economic losses and project temporal cost trends from 1990 to 2025. We hypothesize that (i) climate change will drive higher-latitude expansion of suitable habitats for all four species; and (ii) economic losses associated with these species will rise over time. This integration of habitat suitability modeling and economic loss assessment enables a more comprehensive understanding of the impacts caused by aquatic invasive plants. Enhanced monitoring and management are essential to mitigate associated threats.

## 2 Methods

### 2.1 Species distribution modeling

Species occurrence data for *P. crassipes*, *P. stratiotes*, *A. philoxeroides*, and *C. caroliniana* were obtained from the Global Biodiversity Information Facility (GBIF, https://www.gbif.org/), complemented by published literature and regional surveys. After collecting all occurrence coordinates, Google Earth (https://www.gps-coordinates.net/) was adopted for geographic registration of distribution points. We sorted, cleaned and screened each record, removing duplicate and suspicious data through two complementary approaches: manual point-by-point inspection and duplicate elimination via ENMtools. Global occurrence data were plotted on a world map for visual data validation. To capture the full climatic niche of each species, occurrence records from both native and invaded ranges were pooled for model calibration. This approach provides a more complete representation of the species’ fundamental niche and is widely recommended for predicting potential invasion risk under climate change scenarios.The final cleaned datasets comprised 4,683, 8,843, 11,632, and 2,644 occurrence points, respectively. Species distribution modeling was performed using the Biomod2 ensemble platform (Thuiller et al., 2009) due to its ability to integrate multiple algorithms, reduce single-model bias, and provide weighted average consensus forecasts, which improves predictive robustness compared to single-model approaches (Araújo and New, 2007).

Bioclimatic variables were obtained from the WorldClim database at 2.5 arc-minute resolution. Environmental variables in this study include both bioclimatic variables and non-climatic factors (elevation, population density, NDVI, UVB radiation, land use). For future projections, we used the Coupled Model Intercomparison Project Phase 6 (CMIP6) data. Future projections included nine Shared Socioeconomic Pathway (SSP) scenarios: low (SSP126), medium (SSP245), and high (SSP585) emissions for the years 2030, 2050, and 2070. All data were processed at 2.5 arc-minute resolution (~21 km²at the equator) to balance computational feasibility and ecological relevance, as this resolution captures broad-scale climatic gradients while minimizing noise from fine-scale geographic anomalies (Guisan et al., 2014; Kumar et al., 2009). To minimize multicollinearity among predictor variables, we used ENMtools to calculate pairwise Pearson correlation coefficients and combined MaxEnt contribution evaluation for rigorous variable screening. For variable pairs with |r| > 0.8, only the variable with higher permutation contribution was retained. This two-step screening procedure guaranteed that only ecologically meaningful predictors were incorporated into the final ensemble modeling framework.

Non-climatic variables were selected based on known ecological drivers of aquatic plant invasions (Macêdo et al., 2024; Mouta et al., 2023). These included: elevation (DEM from WorldClim), population density (LandScan 2020), Normalized Difference Vegetation Index (NDVI from NASA MODIS), annual UVB radiation (glUV platform), and land use (ESA CCI-LC). Future scenarios for non-climatic variables were not available; therefore, these variables were held constant at current conditions in future projections, representing a worst-case assumption that human population density and land use patterns remain unchanged.

All environmental layers were resampled to 2.5 arc-minute resolution, cropped to the global land mask (excluding Antarctica), and projected to WGS84 coordinate system using ArcMap 10.8.1 for consistency. In the SDM workflow, both climatic and non-climatic raster layers were used as predictors. Pseudo-absence points (10,000 per species) were randomly generated globally, excluding known occurrence cells. For each species, 80% of occurrence and pseudo-absence data were used for training and 20% for validation (Li and Wang, 2013). The modeling procedure was repeated ten times to account for stochasticity in pseudo-absence selection and data partitioning.

We implemented five algorithms within Biomod2: Generalized Additive Models (GAM, R package mgcv), Generalized Boosted Models (GBM, gbm), Random Forest (RF, randomForest), Classification Tree Analysis (CTA, rpart), and Maximum Entropy (MaxEnt, maxnet). The choice of five models balances computational efficiency with algorithm diversity, covering both regression and machine learning approaches commonly used in invasion SDMs. Ensemble forecasts were generated using a weighted average approach, where weights were proportional to the True Skill Statistic (TSS) scores of individual models. The five models with the highest TSS among the ten replicates were selected for ensemble integration (Thuiller et al., 2009).

Model performance was evaluated using TSS and the Area Under the Receiver Operating Characteristic Curve (ROC/AUC). TSS values >0.7 were considered good, >0.8 excellent, and >0.9 outstanding. ROC values >0.9 indicated high discriminatory ability. Spatial shifts in suitability were quantified by comparing differences in suitable habitat areas (habitat suitability was classified into non-suitable, low-, moderate- and high-suitability zones using the Natural Breaks (Jenks) method) between historical (pre-2000 baseline) and future scenarios, as well as by calculating the centroid movement of high-suitability areas across periods (not solely based on area differences). The predictive performance of ensemble models and the top important variables for each species are summarized in **Supplementary Table 3**.

All modeling procedures were conducted using R version 4.2.2, and additional spatial analyses and map visualizations were performed using ArcMap version 10.8.1.

### 2.2 Economic cost projection

The invacost R package (Leroy et al., 2022) was utilized to analyze the global economic losses caused by invasive aquatic plant species. The analysis was based on the InvaCost 4.1 database (Diagne et al., 2020b), which compiles standardized cost data related to biological invasions. Four invasive aquatic plant species—*P. stratiotes*, *P. crassipes*, *A. philoxeroides*, and *C. caroliniana*—were selected for economic impact assessment. The Invacost 4.1 dataset was imported into R and preprocessed by removing records with missing values. Cost entries corresponding to the target species were filtered and standardized to 2017 US dollars using the exchange rate and inflation correction provided in the ‘Cost_estimate_per_year_2017_USD_exchange_rate’ column (Diagne et al., 2020a). For cost entries covering multiple years (e.g., a single estimate for a 5-year period), we divided the total cost by the number of years to obtain an annualized value, consistent with the approach in Cuthbert et al. (2021). Annual cost values were log10-transformed to reduce skewness and meet normality assumptions of linear models (Leroy et al., 2022). For model fitting and high-reliability estimates, only records with a Method reliability rating of ‘High’ and an Implementation category of ‘Observed’ were retained. Further filtering was conducted using the invacost.NA$Species object to extract cost data specific to the four selected species. The summarize Costs() function was employed to aggregate economic cost data from 1990 onward. Summary tables were generated to display total costs, average annual costs, number of cost estimates, and the number of years represented. The mean annual cost was calculated by summing the total annual costs across each decade and dividing by the number of years in that period, providing a clearer view of long-term economic burdens.

To investigate temporal trends in economic costs, a range of statistical models—including ordinary least squares (OLS), linear regression, quadratic regression, generalized additive models (GAMs), and quantile regression—were applied. These models were chosen because they capture different aspects of temporal dynamics: linear/robust models identify monotonic trends, GAM and MARS detect non-linear patterns and breakpoints, and quantile regression assesses heterogeneity across cost distributions. These models were used to fit time-series data and generate graphical representations of cost trajectories over time. Importantly, the fitted models were used to describe historical trends and extrapolate future costs under the assumption that observed temporal patterns continue. The models do not directly incorporate SDM outputs (e.g., future suitable habitat area) as predictors because no established theoretical relationship exists between habitat suitability and economic cost magnitude. Instead, future cost estimates are based purely on time as a proxy for unobserved social-ecological drivers (e.g., globalization, trade volume, management effectiveness). Through predictive modeling, temporal dynamics of annual invasion-related costs were revealed. Overall, this study employed a comprehensive framework that integrates global-scale economic cost analysis and time-series modeling to assess the financial impacts of invasive aquatic plant species.

## 3 Results

### 3.1 Global suitable areas under current climate conditions

The environmental variables were preprocessed and subsequently evaluated using the jackknife test to determine their relative importance. The variables with the highest contributions were identified as Bio01 (Annual Mean Temperature), Bio02 (Mean Diurnal Range), Bio03 (Isothermality), Bio04 (Temperature Seasonality), Bio11 (Mean Temperature of Coldest Quarter), Bio12 (Annual Precipitation), Bio14 (Precipitation of Driest Month), Bio15 (Precipitation Seasonality), Bio16 (Precipitation of Wettest Quarter), and Bio19 (Precipitation of Coldest Quarter). Based on model performance metrics, the five best-performing algorithms were selected for ensemble modeling: Generalized Additive Models (GAM), Generalized Boosted Models (GBM), Random Forest (RF), Classification Tree Analysis (CTA), and Maximum Entropy (MaxEnt). Model validation results (TSS and ROC scores) for each species are reported below.

**Figure 1:**
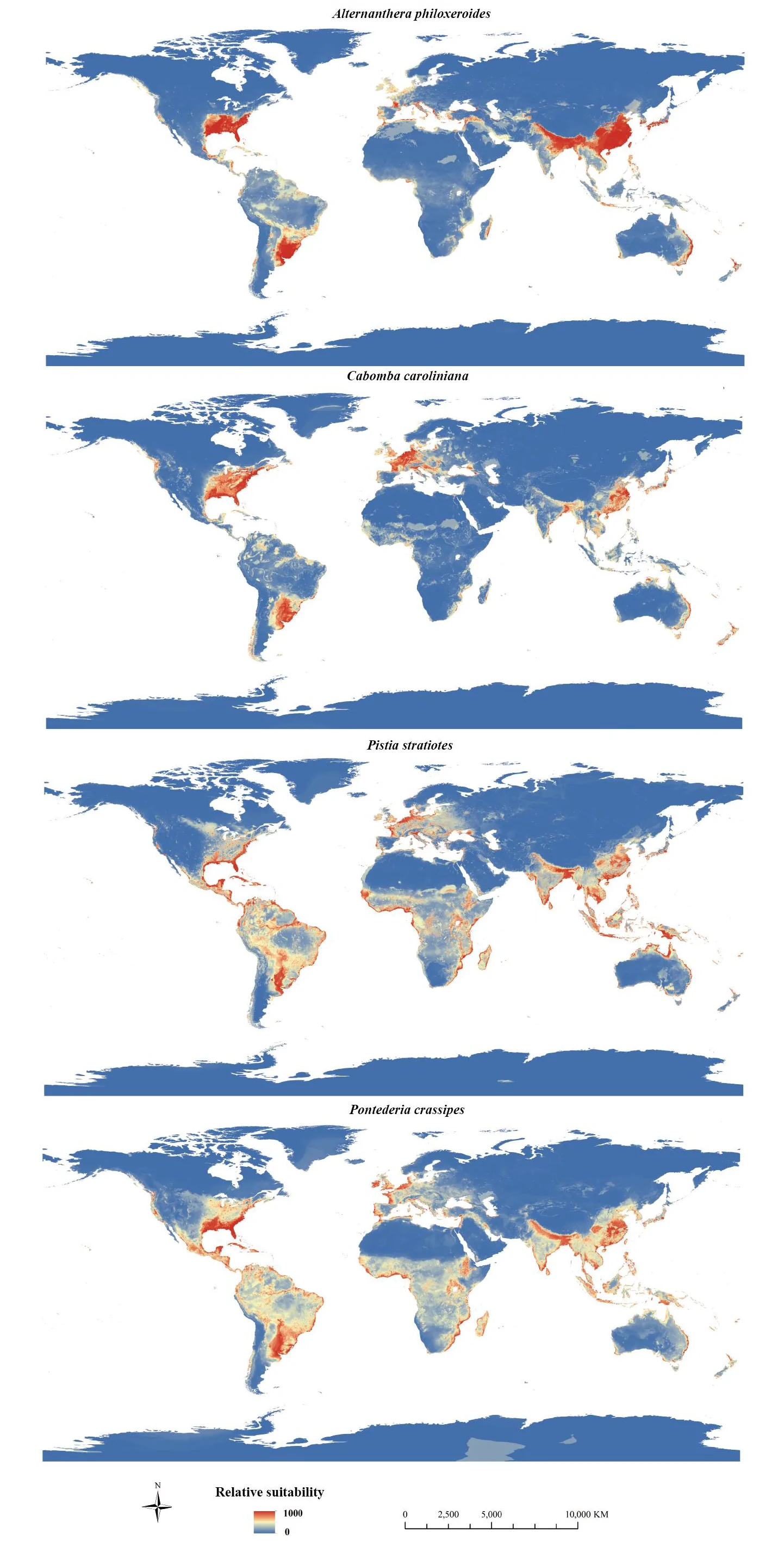
Potential suitable areas for *P. crassipes, P. stratiotes, A. philoxeroides, C. caroliniana* under current climate conditions. Potential suitable habitats of *Alternanthera philoxeroides, Cabomba caroliniana, Pistia stratiotes* and *Pontederia crassipes* under current climate conditions, and potential suitable habitats of Alternanthera philoxeroides under future climate scenarios. Red indicates high-suitability areas, yellow indicates low-suitability areas, and blue indicates unsuitable areas.

For *P. crassipes*, the ensemble model achieved a TSS of 0.818 and an ROC of 0.967, indicating high predictive performance. The most important predictors were Bio01 (Annual Mean Temperature), UVB (Annual UV Radiation), DEM (Elevation), POP (Population Density), and Bio03 (Isothermality). Current high-suitability habitats (habitat suitability was classified into non-suitable, low-, moderate- and high-suitability zones using the Natural Breaks (Jenks) method) for *P. crassipes* are extensive in South Asia, parts of Europe, Oceania, Africa, and low- to mid-latitude areas in North and South America (**Figure 1**). Statistically, the model identified a total of 14.2 million km²of high-suitability area globally under current conditions.

For *P. stratiotes*, the ensemble model produced an ROC score of 0.954. The most influential variables were POP, DEM, Bio01, Bio12, Bio11, Bio04, and Bio03. High-suitability regions under current climate conditions are mainly distributed in South Asia, tropical and subtropical zones of South and North America, parts of Africa, and Europe (**Figure 1**). Total high-suitability area was estimated at 12.8 million km².

For *A. philoxeroides*, the ensemble model achieved a TSS of 0.965 and an ROC of 0.997, indicating excellent model performance. The key contributing variables were Bio01, Bio11, and POP. Under current climatic conditions, the predicted suitable areas for *A. philoxeroides* include South Asia, the southeastern United States, and extensive parts of South America. Europe and Africa showed relatively low suitability (**Figure 1**). Total high-suitability area was 16.1 million km².

For *C. caroliniana*, the model achieved a TSS of 0.946 and an ROC of 0.997. The most important predictors were Bio01, Bio12, and DEM. The predicted suitable areas under current climate conditions are primarily concentrated in South and Southeast Asia, the southeastern United States, and southeastern South America (**Figure 1**). Compared to *P. stratiotes* and *P. crassipes*, the predicted distribution range for *C. caroliniana* was relatively narrower, with a total high-suitability area of 3.9 million km².

### 3.2 Global suitable areas under future climate conditions

**Figure 2:**
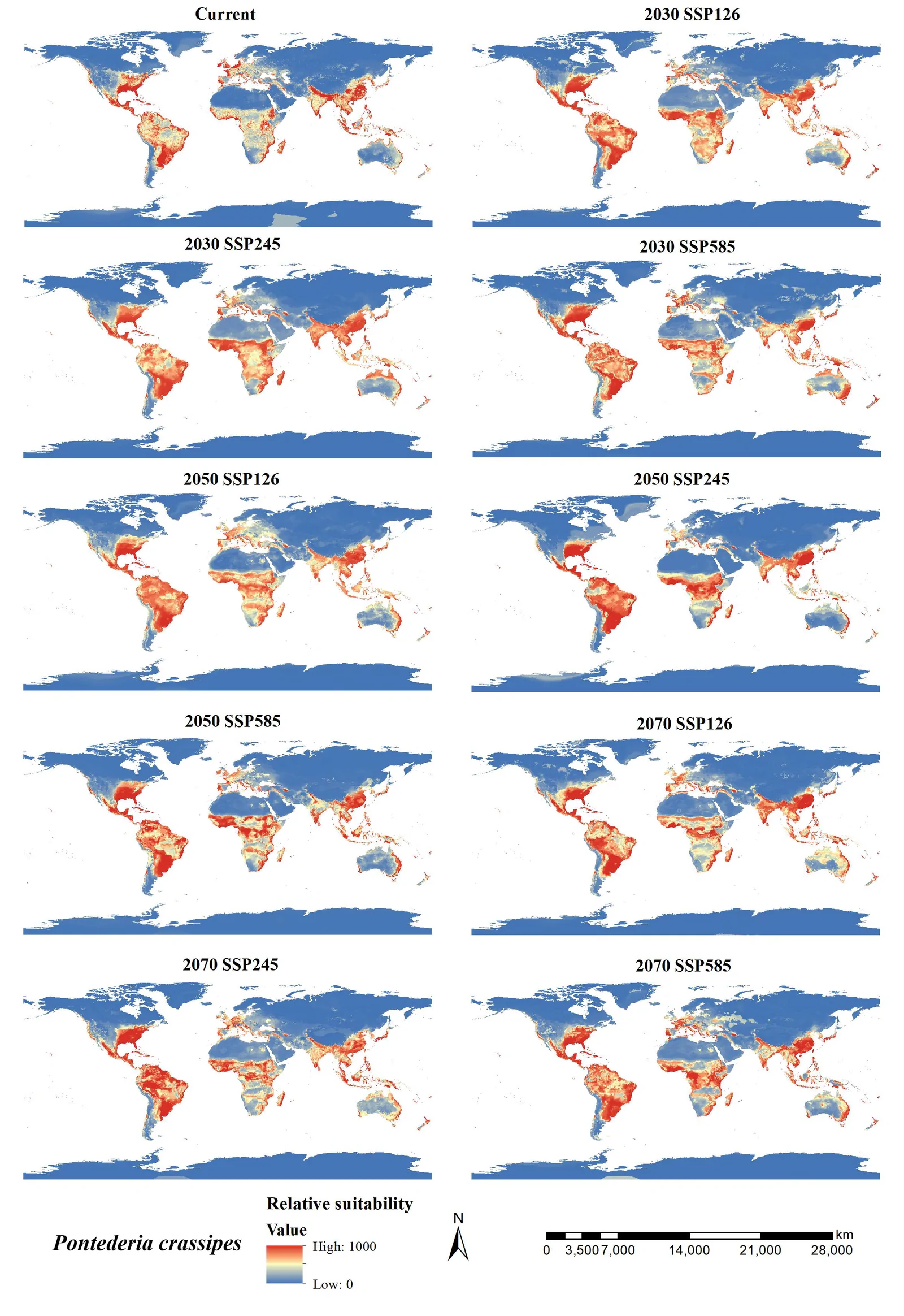
Suitable areas for *P. crassipes* under future climate conditions. Nine future climate scenarios were adopted, generating 36 model outputs in total for the four species. Projections cover three time periods: near-term (2030), mid-term (2050), and long-term (2070). The Shared Socioeconomic Pathways (SSPs) represent low-(SSP126), moderate-(SSP245), and high-emission (SSP585) scenarios.

**Figure 3:**
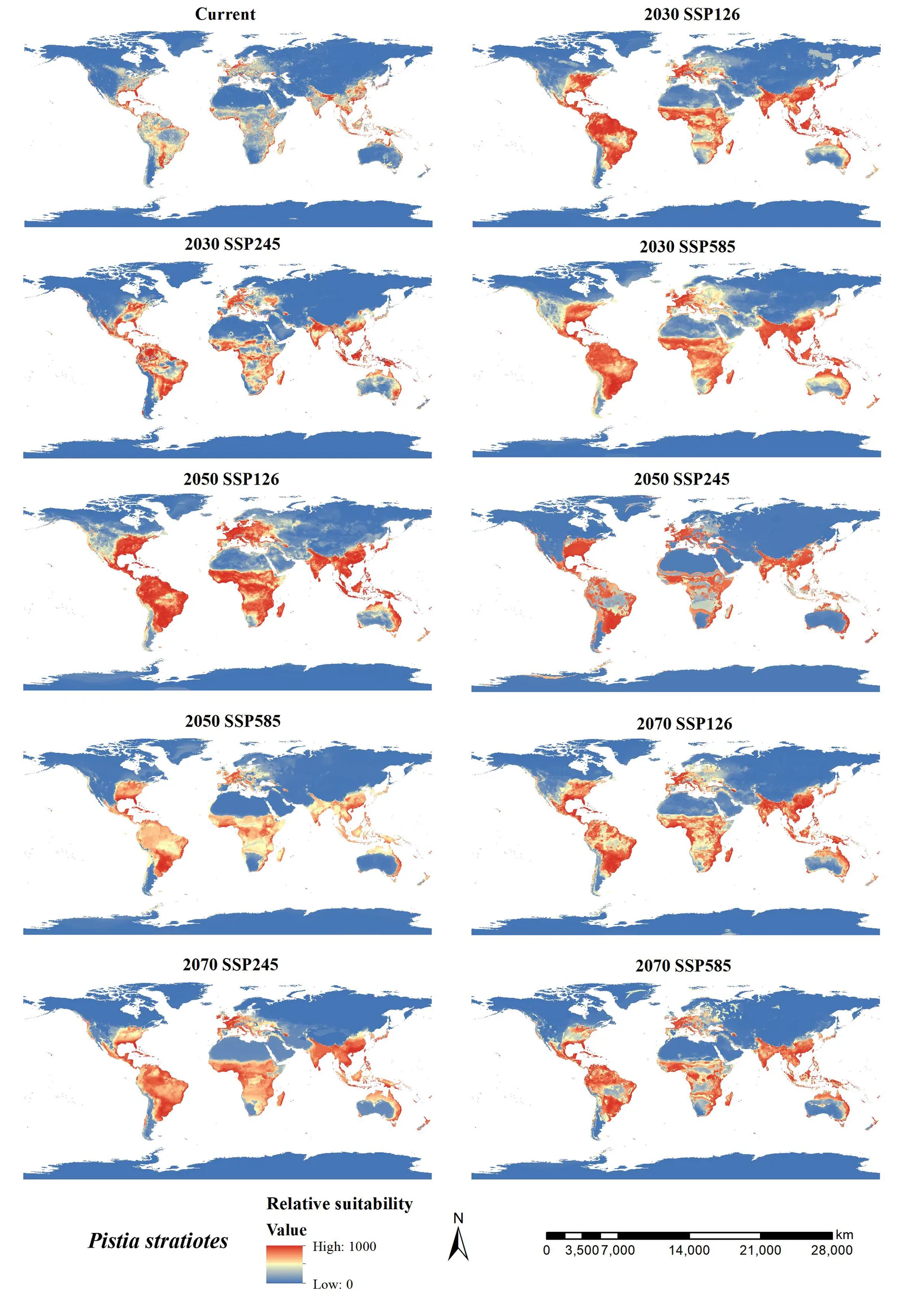
Suitable areas for *P. stratiotes* under future climate conditions. Nine future climate scenarios were adopted, generating 36 model outputs in total for the four species. Projections cover three time periods: near-term (2030), mid-term (2050), and long-term (2070). The Shared Socioeconomic Pathways (SSPs) represent low-(SSP126), moderate-(SSP245), and high-emission (SSP585) scenarios.

**Figure 4:**
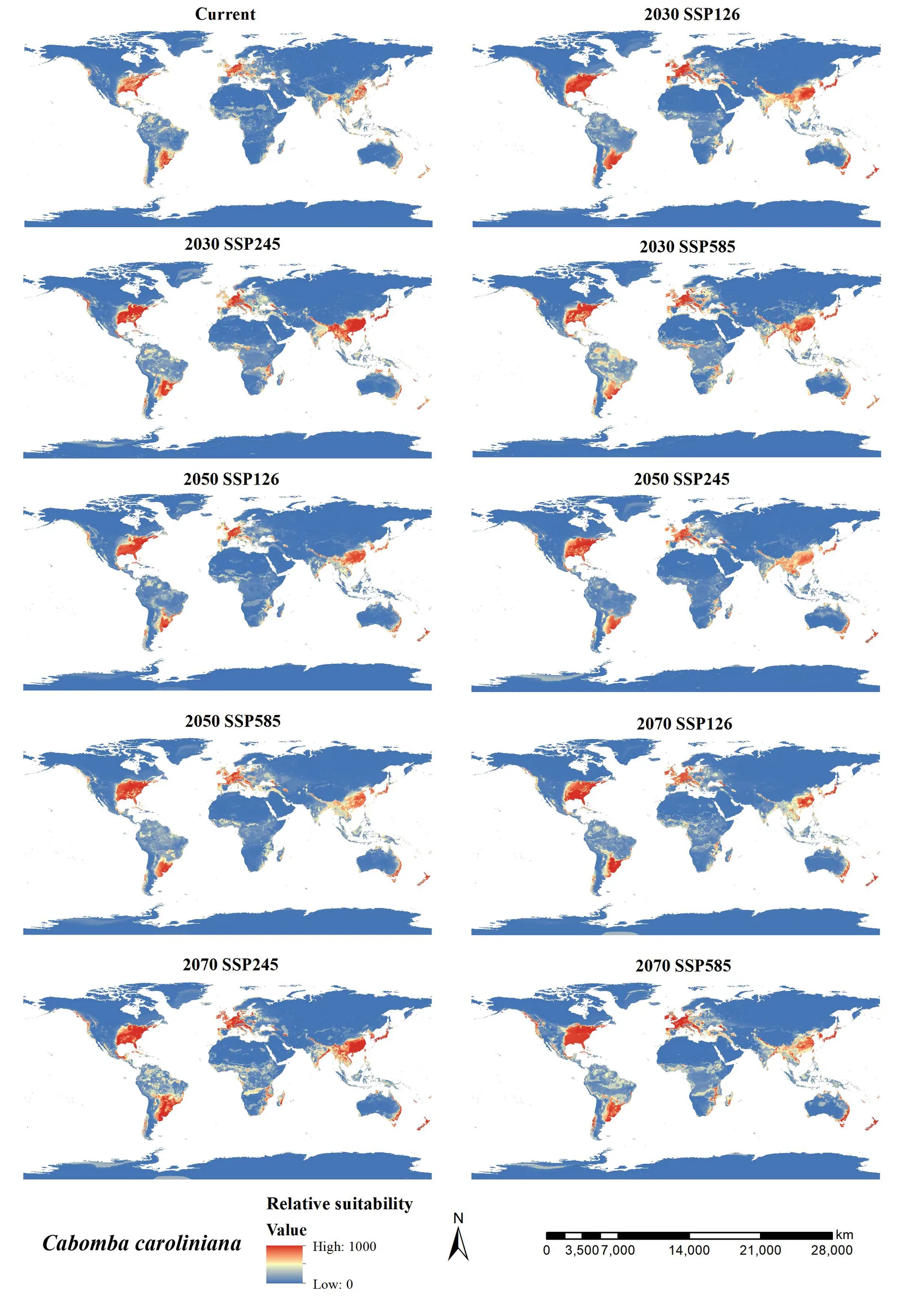
Suitable areas for *C. caroliniana* under future climate conditions. Nine future climate scenarios were adopted, generating 36 model outputs in total for the four species. Projections cover three time periods: near-term (2030), mid-term (2050), and long-term (2070). The Shared Socioeconomic Pathways (SSPs) represent low-(SSP126), moderate-(SSP245), and high-emission (SSP585) scenarios.

**Figure 5:**
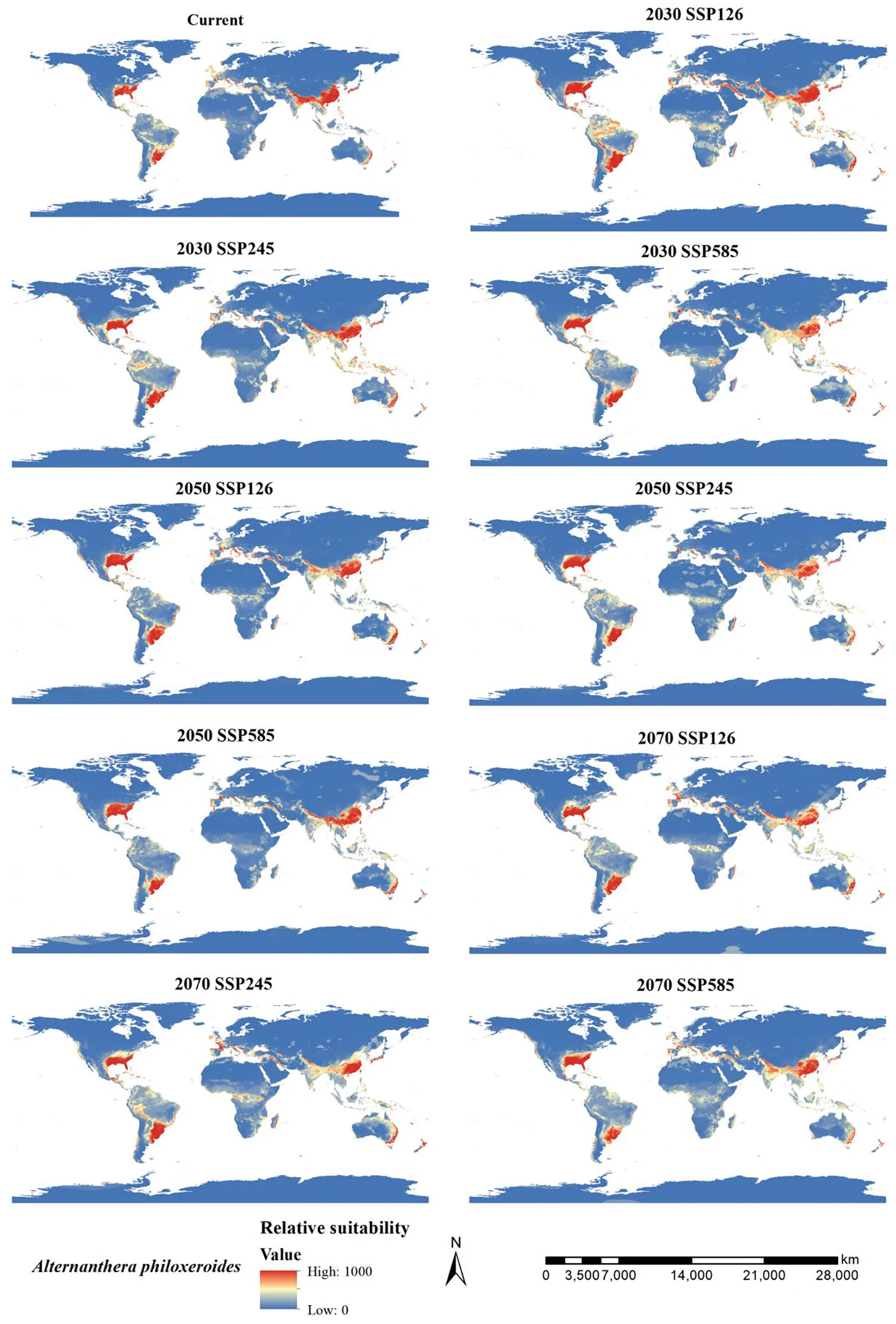
Suitable areas for *A. philoxeroides* under future climate conditions. Nine future climate scenarios were adopted, generating 36 model outputs in total for the four species. Projections cover three time periods: near-term (2030), mid-term (2050), and long-term (2070). The Shared Socioeconomic Pathways (SSPs) represent low-(SSP126), moderate-(SSP245), and high-emission (SSP585) scenarios.

This study predicted the global suitable habitat ranges for four invasive aquatic plant species under current and future climate scenarios. For each species and scenario, we generated one ensemble forecast by averaging the predictions of the five best-performing models (as described in Methods 2.1), yielding a total of 36 ensemble model outputs (4 species×9 scenarios). The predicted suitable habitat distributions are illustrated in **Figures 2**–**5**. Across most scenarios and species, a general expansion of high-suitability areas was observed, indicating potential future increases in habitat suitability under climate change.

**Figure 6:**
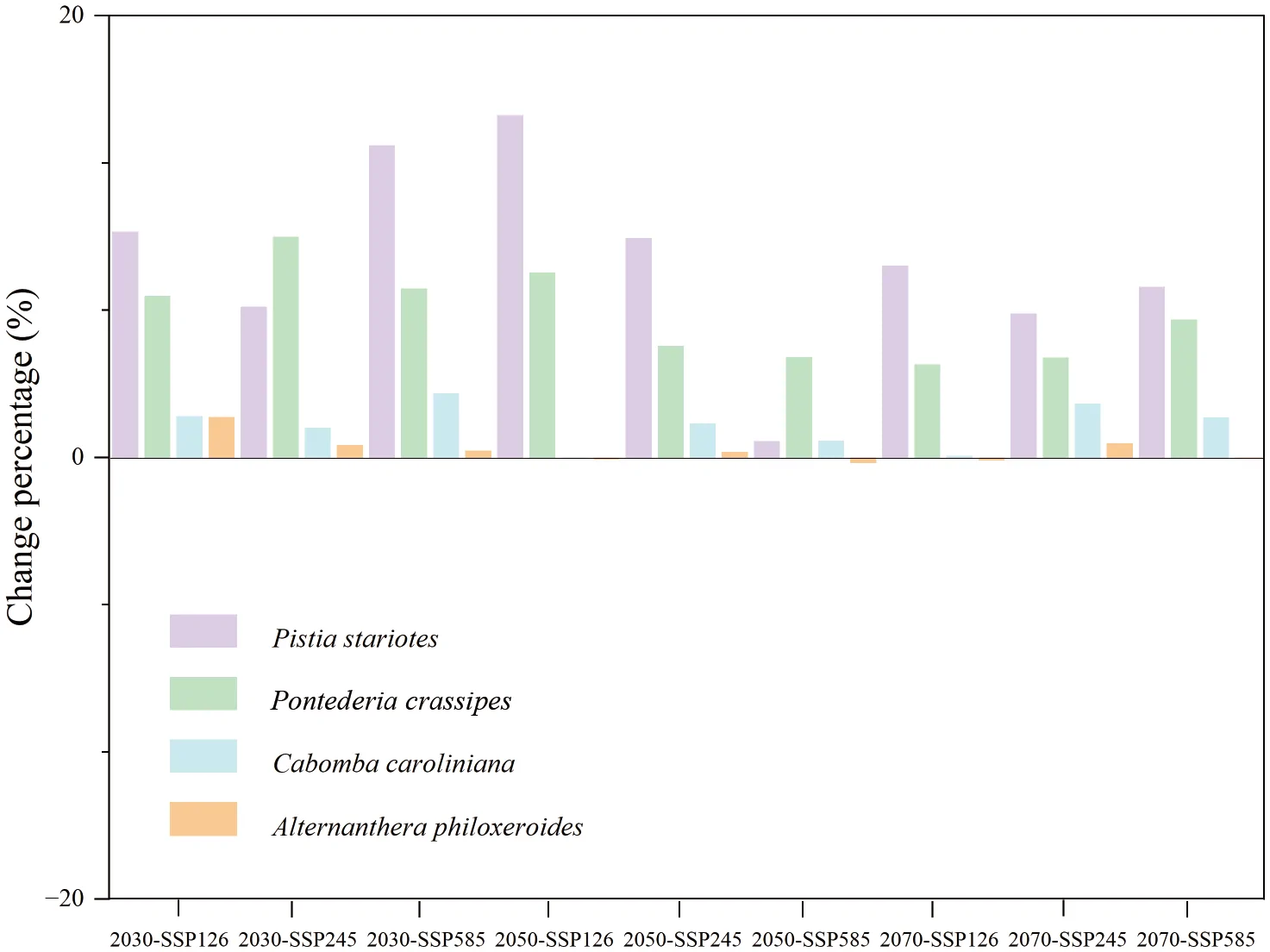
Change percentage for global highly suitable areas of four aquatic plant species under future climate conditions. Percentage change in global highly suitable areas for four aquatic invasive plant species under different future climate scenarios. Nine future climate scenarios were adopted, generating 36 model outputs in total for the four species. Projections cover three time periods: near-term (2030), mid-term (2050), and long-term (2070). The Shared Socioeconomic Pathways (SSPs) represent low-(SSP126), moderate-(SSP245), and high-emission (SSP585) scenarios.

For *P. crassipes*, habitat suitability was predicted to increase under all nine future scenarios, with an expansion range between +4.24% and +9.99%, and an average increase of 6.44% across scenarios (**Figures 2**, **6**). *P. stratiotes* showed moderate to high expansion in most scenarios. Although a minor increase of only +0.74% was observed under the 2050-SSP585 scenario, the remaining scenarios exhibited expansions between +6.28% and +10.21%, with an average increase of 8.86% (**Figures 3**, **6**). *C. caroliniana* exhibited a slight contraction (-0.03%) under the 2050-SSP126 scenario, while all other scenarios showed modest expansion ranging from +0.06% to +2.94%, with a mean increase of 1.41% (**Figures 4**, **6**). For *A. philoxeroides*, habitat expansion was observed in all three scenarios for the year 2030 (increases of +1.82%, +0.56%, and +0.31% under SSP126, SSP245, and SSP585, respectively). Moderate increases were also predicted under 2050-SSP245 and 2070-SSP245 (+0.25% and +0.63%, respectively). In contrast, slight contractions occurred under the remaining scenarios, ranging from -0.25% to -0.03%. The average percent change across all scenarios was +0.34% (**Figures 5**, **6**). In comparison, *P. stratiotes* and *P. crassipes* exhibited the most pronounced expansion in future suitable areas, with average increases between 5% and 10%. *C. caroliniana* showed limited range expansion, while *A. philoxeroides* demonstrated mixed responses, with both expansions and contractions ranging from -0.25% to +1.82% depending on the scenarios. Overall, the projections suggest an expansion of suitable habitats for invasive aquatic plants under future climate conditions, accompanied by a general poleward shift toward higher latitudes.

### 3.3 Global economic losses caused by four invasive aquatic plants

**Figure 7:**
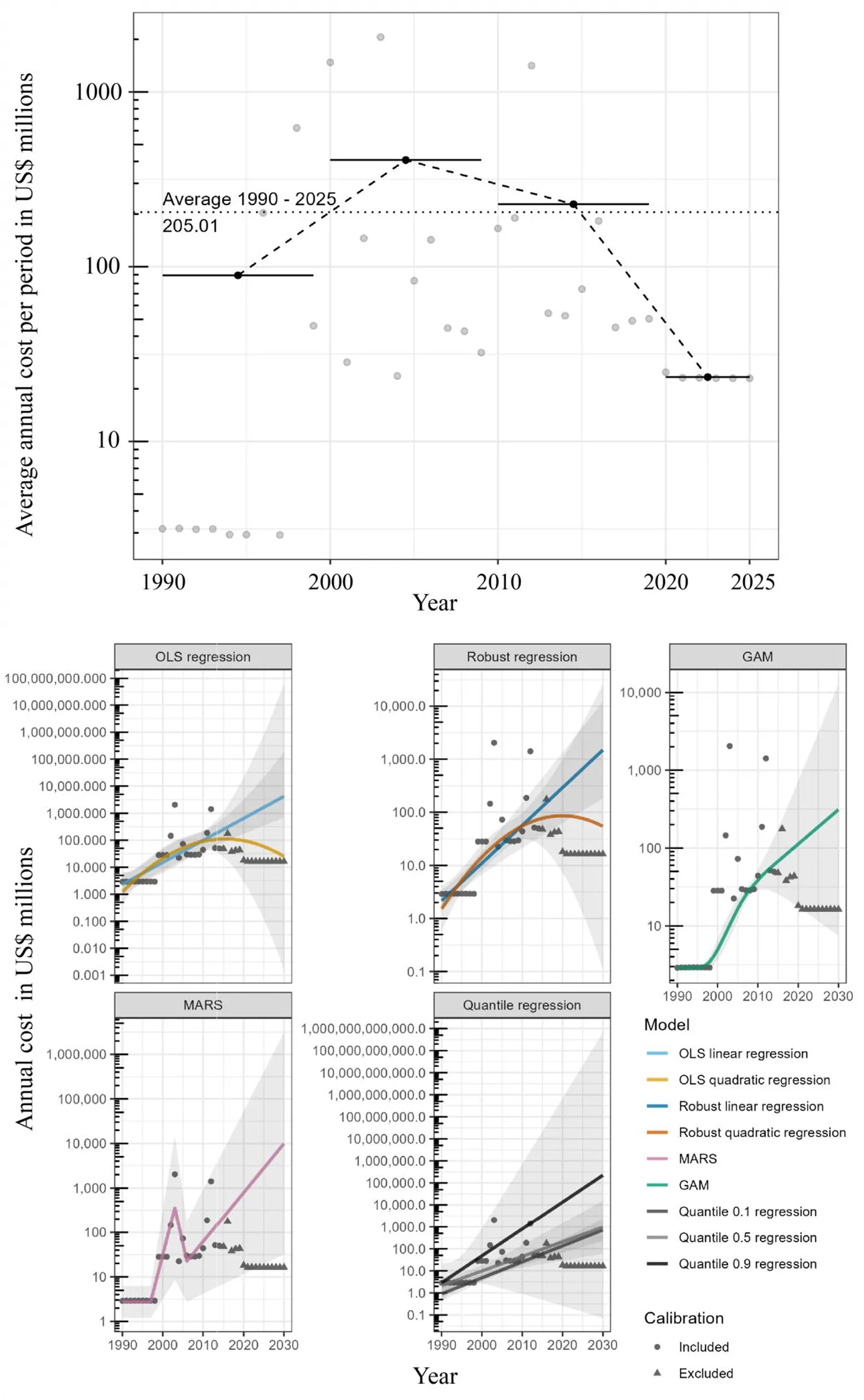
Global economic costs time trend and model fit plots (a. Global economic costs time trend; **(b)** Global economic costs model fit plots.)The panel of global economic costs time trend illustrates the temporal distribution of cost data from 1990 to 2025 to intuitively reflect temporal changes in economic losses. The panel of global economic costs model fit plots shows model-fitting results. Raw cost data were converted to millions of US dollars and then log-transformed. Linear regression, quadratic regression, robust regression, generalized additive model (GAM), Multivariate Adaptive Regression Splines (MARS), and Quantile Regression were used to predict the average annual economic losses caused by invasive species.

Based on filtered and aggregated data from 1990 to 2025, the cumulative reported economic loss attributed to the four species combined amounted to USD 7,380.30 million, with an average annual cost of USD 205.01 million (**Figure 7a**). However, these aggregated global totals should be interpreted with caution, as costs are incurred locally by different sectors and regions, and summing across species and locations may obscure important contextual details. Temporal patterns reveal a peak in annual costs during the early 2000s, followed by a notable decline after approximately 2015 (**Figure 7a**). Between 2000 and 2009, total reported costs reached USD 4,072.71 million (annual average USD 407.27 million), whereas from 2010 to 2019 the total was USD 2,276.34 million (annual average USD 227.63 million). Post-2015 data show a continued downward trajectory, which may reflect improved management, changes in reporting practices, or data gaps.

To examine historical trends, we fitted multiple statistical models to the annual time series (**Figure 7b**). Linear regression indicated a significant but weak upward trend (Re=0.34, *p* = 0.002), while robust regression (Rgressio *p* = 0.005) suggested that outliers influenced the ordinary least squares estimate. A generalized additive model (GAM) captured non-linear patterns (adjusted Radjuste deviance explained=52%), revealing the early-2000s peak and subsequent decline. The low Rowline. across all models indicate that time alone explains less than half of the variance in annual costs, underscoring the influence of unmeasured drivers such as management expenditure, trade volumes, and extreme weather events.

Given the post-2015 decline, the weak explanatory power of the models, and the high uncertainty inherent in extrapolating beyond the observed data range, we refrain from producing quantitative future cost projections. Previous attempts (*e.g.*, quadratic, MARS, and quantile regression models) yielded divergent estimates ranging from USD 25 million to over USD 200 billion for 2030io spread spanning four orders of magnitude that renders any single projection uninformative for policy or management. Instead, we conclude that historical economic losses from these four invasive aquatic plants have been substantial, with a peak in the early 2000s followed by a decline. Future research should move beyond time-only extrapolation by integrating mechanistic drivers (*e.g.*, climate change, trade, management effectiveness) and should avoid aggregating costs across species and regions without clear justification. Improved data collection, particularly post-2020 and for underrepresented regions, is urgently needed to support robust trend analysis.

## 4 Discussion

### 4.1 Drivers of current habitat suitability for invasive aquatic plants

The results under current climatic conditions demonstrate that the ensemble modeling approach yielded robust predictive performance, consistent with previous studies that employed similar methods (Luo et al., 2017). Among the environmental variables, annual mean temperature (Bio01) was identified as the most influential predictor, followed by mean temperature of the coldest quarter (Bio11), precipitation (e.g., Bio12), and elevation. This finding aligns with studies on invasive Asteraceae species, where suitable habitat under climate change scenarios was found to shift toward higher latitudes, primarily driven by precipitation in the coldest quarter, temperature seasonality, and anthropogenic factors such as population density (Yang et al., 2023). Annual mean temperature is typically highly correlated with latitude, and a plant’s capacity to tolerate extreme high and low temperatures largely determines its latitudinal and altitudinal distribution limits. Climatic factors thus play a dominant role in shaping the global distribution of plant species (Huang et al., 2021). Our study shows that regions of high habitat suitability for invasive aquatic plants are primarily concentrated in the South Asian monsoon zone (e.g., Ganges–Mekong River basins), mid- to low-latitude areas of the Americas (e.g., lower Amazon and Mississippi River), and tropical lake regions of Africa (e.g., Lake Victoria basin). These areas share similar climatic characteristics and are often associated with intensive human activities such as shipping and agricultural irrigation. Such interactions between favorable climate and anthropogenic disturbance contribute to the formation of invasion hotspots, with both environmental and human-driven forces jointly facilitating the establishment and spread of invasive species (Murphy et al., 2022).

### 4.2 Habitat suitability shifts under future climate scenarios

Global climate change is accelerating the process of biological invasions, amplifying both ecological and economic impacts, and highlighting the urgent need for coordinated management (Hulme, 2017). Projections under future climate scenarios reveal an overall expansion in suitable habitat areas for the four aquatic invasive plant species studied, along with a discernible poleward shift in their potential distributions. The most substantial increases in habitat suitability were observed for *P. crassipes* and *P. stratiotes*, with maximum area increases of 9.99% and 10.21%, respectively. These trends are likely driven by rising annual mean temperature (Bio01) and a reduction in extreme low-temperature events in winter (elevated Bio11 values), which have weakened the climatic barriers in higher latitudes.

In contrast, *A. philoxeroides* showed a more modest net change in future suitable habitat area, ranging from –0.25% to 1.82%. This could be attributed to the adverse effects of extreme high temperatures limiting its expansion. *C. caroliniana*, a submerged species, exhibited the smallest habitat expansion, which is likely related to its specific ecological traits. Among the most influential environmental variables identified in our models were Bio11 (mean temperature of the coldest quarter) and Bio04 (temperature seasonality). These climatic variables are known to enhance phenotypic plasticity in plants, thereby affecting their spatial distribution at both global and regional scales (Huang et al., 2021). Favorable climatic shifts under future scenarios may thus enable invasive species to colonize new regions, increasing invasion risk (Bradley et al., 2010).

Our findings are consistent with previous model-based studies on other invasive species such as *Parthenium hysterophorus* and *Reynoutria japonica*, which have also shown significant poleward expansions in suitable habitats driven by changes in annual temperature and precipitation patterns (Zhang et al., 2024; Zheng et al., 2024). Similar conclusions have been drawn from studies using MaxEnt and ensemble modeling approaches, indicating that climate change will likely facilitate the spread of many invasive species and exacerbate their ecological and economic impacts (Feng et al., 2024; Zhang et al., 2024). However, it is important to note that our models do not incorporate biological characteristics such as dispersal ability, reproduction rate, or interspecific competition. Future expansions may be modulated by these traits, and the projected poleward shifts should be considered potential rather than deterministic. The synergistic global effects of climate change and biological invasion are reshaping species distribution patterns (Chen et al., 2011), thereby altering ecosystem vulnerability across biogeographic regions. Biodiversity hotspots are increasingly subjected to the dual pressures of warming (increased Bio01) and expanding suitable ranges of invasive species, potentially leading to niche compression in native aquatic communities. The explosive spread of *P. crassipes*, for instance, may intensify eutrophication and threaten local fisheries due to its rising suitability in previously uninhabitable regions (Wu and Ding, 2019). Moreover, the projected northward expansion of suitable areas underscores the potential for new invasion events in currently unaffected regions.

### 4.3 Temporal trends and model-based insights into economic impacts

Our economic loss analysis reveals that the period from 2000 to 2019 was the most severe in terms of economic damage caused by the four invasive aquatic plant species. This trend is likely associated with accelerated globalization, increased international trade, and climate change—factors known to facilitate the spread and establishment of invasive species (Cuthbert et al., 2021, 2022). The sharp increase in economic impacts in the early 21st century may also reflect improvements in monitoring and reporting mechanisms. The decline in average annual costs after 2020 may indicate the strengthening of management interventions or limitations in data availability and estimation methods. Across the dataset, damage-related expenditures far exceeded management costs, aligning with previous region-specific findings that proactive management remains insufficient (Crystal-Ornelas et al., 2021; Kourantidou et al., 2022; Liu et al., 2021). This underscores a systemic bias toward reactive responses to invasions rather than preventive strategies.

Our analysis further explored temporal dynamics of annual economic costs using multiple statistical approaches. Consistent with global trends reported in large-scale assessments (Diagne et al., 2021a; Turbelin et al., 2023), we observed a peak in reported costs during the early 2000s followed by a decline after 2015. Linear and robust regression models captured a weak but statistically significant increasing trend over the full period, but this was driven largely by the low-cost years before 2000 and the high-cost years in the early 2000s. Generalized additive models (GAM) revealed pronounced non-linearity, with explained deviance of only 52%, indicating that temporal trends alone cannot reliably predict future costs. The post-2015 decline may reflect improved management interventions, changes in reporting completeness, or limitations in the Invacost database’s coverage for recent years. Critically, the low Ro values (aluesa across all models imply that unmeasured driversred,s as international trade volumes, climate extremes, and control effortls,on substantial roles. Given these limitations and the extreme divergence of extrapolated estimates (spanning orders of magnitude), we consider quantitative future cost projections from time-only models to be unreliable. Future research should integrate ecological, socioeconomic, and climatic covariates into multivariate frameworks rather than relying on temporal extrapolation, and should avoid aggregating costs across species and regions without explicit justification.

### 4.4 Implications for invasion risk management under climate change

Investigating the impacts of climate change on plant distribution and biological invasions enhances our understanding of species’ ecological responses to climate variability, and is essential for addressing global-scale challenges such as predicting future invasion hotspots. There is an urgent need to develop a multi-scale prevention and control framework that integrates both ecological insights and policy implementation (Hulme et al., 2008).

In regions with high habitat suitability—such as Southeast Asia and South America—regional cooperation mechanisms should be strengthened, with an emphasis on the deployment of real-time monitoring networks supported by remote sensing technologies (Yemshanov et al., 2019). In mid- to low-suitability areas, including high-latitude regions such as Northern Europe and North America, restoration of riparian buffer zones can mitigate the spread of aquatic invasive species by reducing nutrient runoff and creating physical barriers to propagule dispersal (Mouta et al., 2023). Future research should incorporate global trade data and extreme climate scenario modeling to quantify the interactive effects of anthropogenic activity and climate extremes on habitat suitability dynamics. Such integration would be instrumental in improving early warning systems for biosafety risks (Hulme, 2017), allowing for more proactive and spatially targeted management interventions.

### 4.5 Limitations and future directions

Despite providing valuable insights, this study has certain limitations. One notable constraint lies in the limited temporal and spatial coverage of economic cost data, particularly for the period after 2020. Incomplete or underreported data from certain countries and regions may lead to an underestimation of the actual global economic losses associated with invasive aquatic plant species. Future research should aim to enhance data completeness and temporal consistency to enable more comprehensive assessments of economic impacts. It is also essential to focus on identifying and quantifying the underlying drivers of invasive species-related economic costs. Integrating socioeconomic and ecological datasets will allow for a more nuanced exploration of how factors such as climate change, international trade, and land-use change contribute to economic impacts (Wang et al., 2023). This approach can provide a scientific basis for designing targeted and context-specific management strategies.

Furthermore, efforts should be made to strengthen data collection and international data-sharing mechanisms. Enhanced global collaboration—particularly focused on data-deficient regions, including many developing countries—would improve the geographic coverage and accuracy of cost estimations (Crystal-Ornelas et al., 2021; Diagne et al., 2021b, 2020a). Establishing open-access and standardized databases will facilitate timely information exchange and cross-national comparisons. Evaluating the economic effectiveness of existing control measures (e.g., biological control, mechanical removal) is also a key research priority. Cost-benefit analyses of these interventions can inform more efficient resource allocation and guide evidence-based policy development. From a policy and management perspective, there is a pressing need to strengthen early detection and rapid response systems to minimize the establishment risk of newly introduced species (Macêdo et al., 2024). Given the transboundary nature of biological invasions, enhanced international cooperation is critical. Developing globally coordinated prevention and management strategies will be essential to mitigate the ecological and economic threats posed by invasive species. By quantifying the economic losses caused by four globally significant invasive aquatic plants, this study contributes to the growing body of evidence highlighting the urgency of addressing biological invasions. Future research should expand species coverage, incorporate more diverse environmental and socioeconomic variables, and promote collaborative monitoring to support global invasive species risk management.

## 5 Conclusions

The global habitat suitability predictions indicate that the ensemble modeling approach demonstrates strong predictive performance. Among the environmental and anthropogenic variables, annual mean temperature, population density, and mean temperature of the coldest quarter were consistently among the most influential predictors across species. Precipitation and elevation contributed less significantly in most models. Spatially, *P. crassipes* is projected to be highly suitable in South Asia, as well as parts of Europe, Oceania, Africa, and mid- to low-latitude regions of the Americas. *P. stratiotes* demonstrates high suitability across South Asia, South and North America, Africa, and parts of Europe. *A. philoxeroides* currently has a broad potential distribution in South Asia, southeastern North America, and most parts of South America, while Europe and Africa remain areas of low suitability. In contrast, *C. caroliniana* exhibits a more restricted suitable area, primarily concentrated in South Asia, Southeast Asia, southeastern North America, and parts of South America. Future climate scenarios suggest a northward shift and spatial expansion in suitable areas for all four species. The most notable increases are seen in *P. crassipes* and *P. stratiotes*, with projected range expansions of 9.99% and 10.21%, respectively. *A. philoxeroides* shows a relatively stable distribution, with changes ranging from -0.25% to +1.82%, while *C. caroliniana* has the smallest expansion potential, likely due to its submerged growth form and ecological preferences.

From an economic perspective, our analysis of the Invacost database revealed substantial global economic losses caused by these four invasive aquatic plant species. Between 1990 and 2025, the total reported economic cost amounted to USD 7,380.30 million, with an average annual cost of USD 205.01 million. Time-series modeling using multiple statistical approaches—including linear regression, robust linear regression, generalized additive models (GAM), and multivariate adaptive regression splines (MARS)—indicated a significant overall upward trend in annual economic losses. Linear and robust regressions showed a weak upward trend over the full period, but GAM revealed a peak in the early 2000s followed by a post-2015 decline, indicating that simple monotonic trends are inadequate. The low explanatory power of all models (Rd ≤ 0.45) and the post-2015 decline suggest that time alone is a poor predictor of future costs. Future research should integrate additional explanatory variables such as climate change, land-use change, and global trade flows in multivariate modeling frameworks, rather than relying on time extrapolation. Expanding the temporal span of cost data and the range of species analyzed, while avoiding unjustified aggregation across species and regions, would also improve model accuracy and generalizability. Enhancing international cooperation and promoting open data shariningonality. with a focus on underrepresented regions in the Global Southlspre be essential for more accurate and equitable global assessments of economic impacts and management priorities.

Our findings offer important insights for predicting global habitat suitability and assessing the economic consequences of invasive aquatic plants. However, further work is needed to explore the mechanistic links between suitable habitat changes and future economic costs, as well as the effectiveness of different management measures. From a policy perspective, strengthening early warning systems and rapid response mechanisms, improving international coordination, and developing real-time monitoring frameworks using remote sensing technologies are all critical. A global, coordinated approach is required to mitigate the escalating ecological and economic risks posed by invasive aquatic plant species in the face of climate change.

## Data Availability

The datasets presented in this study can be found in online repositories. The names of the repository/repositories and accession number(s) can be found in the article/**Supplementary Material**.
