# Supplementary material for: Managing global risks of invasive aquatic plants under climate change: ensemble species distribution modeling and economic cost projections
Source: Front Plant Sci. 2026 Sep 7;17:1895896. doi: 10.3389/fpls.2026.1895896 (PMC13590699; doi:10.3389/fpls.2026.1895896)
Supplement: Supplementary file 1 [file Table1.docx]

**Table S1 Nine future climate scenarios used in this study**

| Climate Scenario | Description |
| --- | --- |
| 2030-SSP126 | 2030 (near-term), low-emission scenario |
| 2030-SSP245 | 2030 (near-term), medium-emission scenario |
| 2030-SSP585 | 2030 (near-term), high-emission scenario |
| 2050-SSP126 | 2050 (mid-term), low-emission scenario |
| 2050-SSP245 | 2050 (mid-term), medium-emission scenario |
| 2050-SSP585 | 2050 (mid-term), high-emission scenario |
| 2070-SSP126 | 2070 (long-term), low-emission scenario |
| 2070-SSP245 | 2070 (long-term), medium-emission scenario |
| 2070-SSP585 | 2070 (long-term), high-emission scenario |

**Table S2 Environmental variables used in model predictions**

| Abbreviations | Variables |
| --- | --- |
| Bio01 | Annual Mean Temperature |
| Bio02 | Mean Diurnal Range |
| Bio03 | Isothermality (Bio02/Bio07) (* 100) |
| Bio04 | Temperature Seasonality |
| Bio05 | Max Temperature of Warmest Month |
| Bio06 | Min Temperature of Coldest Month |
| Bio07 | Temperature Annual Range (Bio05-Bio06) |
| Bio08 | Mean Temperature of Wettest Quarter |
| Bio09 | Mean Temperature of Driest Quarter |
| Bio10 | Mean Temperature of Warmest Quarter |
| Bio11 | Mean Temperature of Coldest Quarter |
| Bio12 | Annual Precipitation |
| Bio13 | Precipitation of Wettest Month |
| Bio14 | Precipitation of Driest Month |
| Bio15 | Precipitation Seasonality |
| Bio16 | Precipitation of Wettest Quarter |
| Bio17 | Precipitation of Driest Quarter |
| Bio18 | Precipitation of Warmest Quarter |
| Bio19 | Precipitation of Coldest Quarter |
| POP | Population density |
| LC | Landcover |
| NDVI | Normalized Difference Vegetation Index |
| UVB | Ultraviolet Radiation B |
| DEM | Digital Elevation Model |

**Table S3 Summary of global suitable habitat predictions for four aquatic invasive plants**

| Item | *P. crassipes* | *P. stratiotes* | *A. philoxeroides* | *C. caroliniana* |
| --- | --- | --- | --- | --- |
| Number of occurrence points | 4,683 | 8,843 | 11,632 | 2,644 |
| Selected bioclimatic variables | BIO01, BIO02, BIO03, BIO04, BIO11, BIO12, BIO14, BIO15, BIO16, BIO19 | BIO01, BIO02, BIO03, BIO04, BIO11, BIO12, BIO14, BIO15, BIO16, BIO19 | BIO01, BIO02, BIO03, BIO04, BIO11, BIO12, BIO14, BIO15, BIO18, BIO19 | BIO01, BIO02, BIO03, BIO04, BIO11, BIO12, BIO14, BIO15, BIO16, BIO19 |
| Non-climatic variables | DEM, POP, UVB, Land cover | DEM, POP, UVB, Land cover | DEM, POP, UVB, Land cover | DEM, POP, UVB, Land cover |
| ROC/AUC | 0.967 | 0.954 | 0.997 | 0.997 |
| Variable importance (top 3) | 1. BIO01 (Annual Mean Temperature)2. UVB (Annual UVB Radiation)3. DEM (Elevation) | 1. POP (Population Density)2. DEM (Elevation)3. BIO01 (Annual Mean Temperature) | 1. BIO01 (Annual Mean Temperature)2. BIO11 (Mean Temperature of Coldest Quarter)3. POP (Population Density) | 1. BIO01 (Annual Mean Temperature)2. BIO12 (Annual Precipitation)3. DEM (Elevation) |
| Future climate scenarios | 2030/2050/2070 × SSP126/SSP245/SSP585, 9 scenarios in total | | | |

**Figure S1**. Cleaned GBIF occurrence points of four aquatic invasive plant species occurrence point counts for modeling: *Alternanthera philoxeroides*(11632), *Cabomba caroliniana*(2644), *Pistia stratiotes*(8843), *Pontederia crassipes* (4683). Occurrence records of four aquatic invasive species were obtained from the Global Biodiversity Information Facility (GBIF). *Pistia stratiotes*: [GBIF.org](https://link.wtturl.cn/?target=https://GBIF.org&scene=im&aid=582478&lang=zh) (29 July 2024) GBIF Occurrence Download, [https://doi.org/10.15468/dl.rxs9a4](https://link.wtturl.cn/?target=https://doi.org/10.15468/dl.rxs9a4&scene=im&aid=582478&lang=zh); *Pontederia crassipes*: [GBIF.org](https://link.wtturl.cn/?target=https://GBIF.org&scene=im&aid=582478&lang=zh) (15 August 2024) GBIF Occurrence Download, [https://doi.org/10.15468/dl.vry6wv](https://link.wtturl.cn/?target=https://doi.org/10.15468/dl.vry6wv&scene=im&aid=582478&lang=zh); *Cabomba carolinian*a: [GBIF.org](https://link.wtturl.cn/?target=https://GBIF.org&scene=im&aid=582478&lang=zh) (29 July 2024) GBIF Occurrence Download, [https://doi.org/10.15468/dl.5b5aqh](https://link.wtturl.cn/?target=https://doi.org/10.15468/dl.5b5aqh&scene=im&aid=582478&lang=zh); *Alternanthera philoxeroides*: [GBIF.org](https://link.wtturl.cn/?target=https://GBIF.org&scene=im&aid=582478&lang=zh) (16 August 2024) GBIF Occurrence Download, [https://doi.org/10.15468/dl.kzzzv5](https://link.wtturl.cn/?target=https://doi.org/10.15468/dl.kzzzv5&scene=im&aid=582478&lang=zh).
